# Observer variability of absolute and relative thrombus density measurements in patients with acute ischemic stroke

**DOI:** 10.1007/s00234-015-1607-4

**Published:** 2015-10-22

**Authors:** Emilie M. M. Santos, Albert J. Yoo, Ludo F. Beenen, Olvert A. Berkhemer, Mark D. den Blanken, Carrie Wismans, Wiro J. Niessen, Charles B. Majoie, Henk A. Marquering, on behalf of the MR CLEAN investigators

**Affiliations:** Department of Radiology, Erasmus MC - University Medical Center Rotterdam, P.O. Box 2040, 3000 CA Rotterdam, The Netherlands; Department of Radiology, AMC, Amsterdam, The Netherlands; Texas Stroke Institute, Plano, TX USA; Department of Neurology, Erasmus MC, Rotterdam, The Netherlands; Department of Biomedical Engineering and Physics, AMC, Amsterdam, The Netherlands; Faculty of Applied Sciences, Delft University of Technology, Delft, The Netherlands

**Keywords:** Brain ischemia, Thromboembolism, Computed tomography, X-ray, Observer agreements

## Abstract

**Introduction:**

Thrombus density may be a predictor for acute ischemic stroke treatment success. However, only limited data on observer variability for thrombus density measurements exist. This study assesses the variability and bias of four common thrombus density measurement methods by expert and non-expert observers.

**Methods:**

For 132 consecutive patients with acute ischemic stroke, three experts and two trained observers determined thrombus density by placing three standardized regions of interest (ROIs) in the thrombus and corresponding contralateral arterial segment. Subsequently, absolute and relative thrombus densities were determined using either one or three ROIs. Intraclass correlation coefficient (ICC) was determined, and Bland–Altman analysis was performed to evaluate interobserver and intermethod agreement. Accuracy of the trained observer was evaluated with a reference expert observer using the same statistical analysis.

**Results:**

The highest interobserver agreement was obtained for absolute thrombus measurements using three ROIs (ICCs ranging from 0.54 to 0.91). In general, interobserver agreement was lower for relative measurements, and for using one instead of three ROIs. Interobserver agreement of trained non-experts and experts was similar. Accuracy of the trained observer measurements was comparable to the expert interobserver agreement and was better for absolute measurements and with three ROIs. The agreement between the one ROI and three ROI methods was good.

**Conclusion:**

Absolute thrombus density measurement has superior interobserver agreement compared to relative density measurement. Interobserver variation is smaller when multiple ROIs are used. Trained non-expert observers can accurately and reproducibly assess absolute thrombus densities using three ROIs.

**Electronic supplementary material:**

The online version of this article (doi:10.1007/s00234-015-1607-4) contains supplementary material, which is available to authorized users.

## Introduction

In acute ischemic stroke, imaging provides information that is crucial to proper patient selection for emergent treatments, including the presence and level of vessel occlusion and the extent of irreversible tissue injury [1]. More recently, thrombus density on non-contrast CT (NCCT) has been shown to be a potential predictor for acute ischemic stroke treatment success [2–5]. Clot density is usually assessed by gross visual inspection (e.g., hyperdense artery sign). Quantitative assessment of thrombus density can be performed by placing one or multiple small regions of interest (ROIs) in the thrombus—for example, using measuring tools available on diagnostic workstations to create ROIs of specific size and shape [5, 6]. Because thrombus density measurements are affected by hematocrit, some groups have measured the density in the corresponding contralateral vessel to calculate a relative density [7]. Four methods have been employed in the recent literature: absolute thrombus density in Hounsfield unit (aHU) and relative thrombus density in Hounsfield unit (rHU) using either one or three ROIs. Little is known regarding the interobserver agreement of these measurements. Furthermore, the impact of expertise and the bias due to using one ROI instead of three ROIs have not been studied.

Our goals were to examine the interobserver agreement of both experts and non-experts for the four common thrombus density measurement methods, and to evaluate the possible bias introduced by using one rather than multiple ROIs.

## Methods

### Patient selection

We collected image data from the Dutch MR CLEAN clinical trial, a multicenter randomized controlled trial that evaluated whether intraarterial treatment of patients with acute ischemic stroke improved functional outcome at 3 months [8]. The main criterion for study inclusion was concurrent thin-slice (≤2.5 mm) NCCT and CT angiography (CTA) image data. As it can be difficult to detect low-density thrombi on NCCT, and standardized CTA imaging is increasingly used as part of standard admission protocol for patients suspected of acute stroke [1], CTA is commonly used for visual support in most thrombus density studies [3–5, 9–11]. In some studies, it was reported that CTA and NCCT were registered for the assessment of the clot burden [12]. We consequently, also used CTA for visual support to localize thrombi. Furthermore, we mandated that the time interval between these scans be a maximum of 30 min because possible IV thrombolysis initiated directly after NCCT acquisition may cause thrombus alteration or migration [1]. We retrospectively collected image data from 388 available patients at the time of this study, of which 204 patients had both thin-slice NCCT and CTA image data. We excluded patients due to time delay between modalities greater than 30 min (*N* = 13), movement artifacts (*N* = 39), extensive noise (*N* = 5), incomplete coverage of the intracranial arteries (*N* = 4), and major differences in thrombus location between the two image modalities (*N* = 2). Out of the remaining 141 patients, 9 were included in the training set, and the other 132 constituted the test set. A table with detailed information about the scanners, CT parameters, and reconstructions parameters can be found in the Online Resource 1.

### Thrombus density measurement

**Fig. 1:**
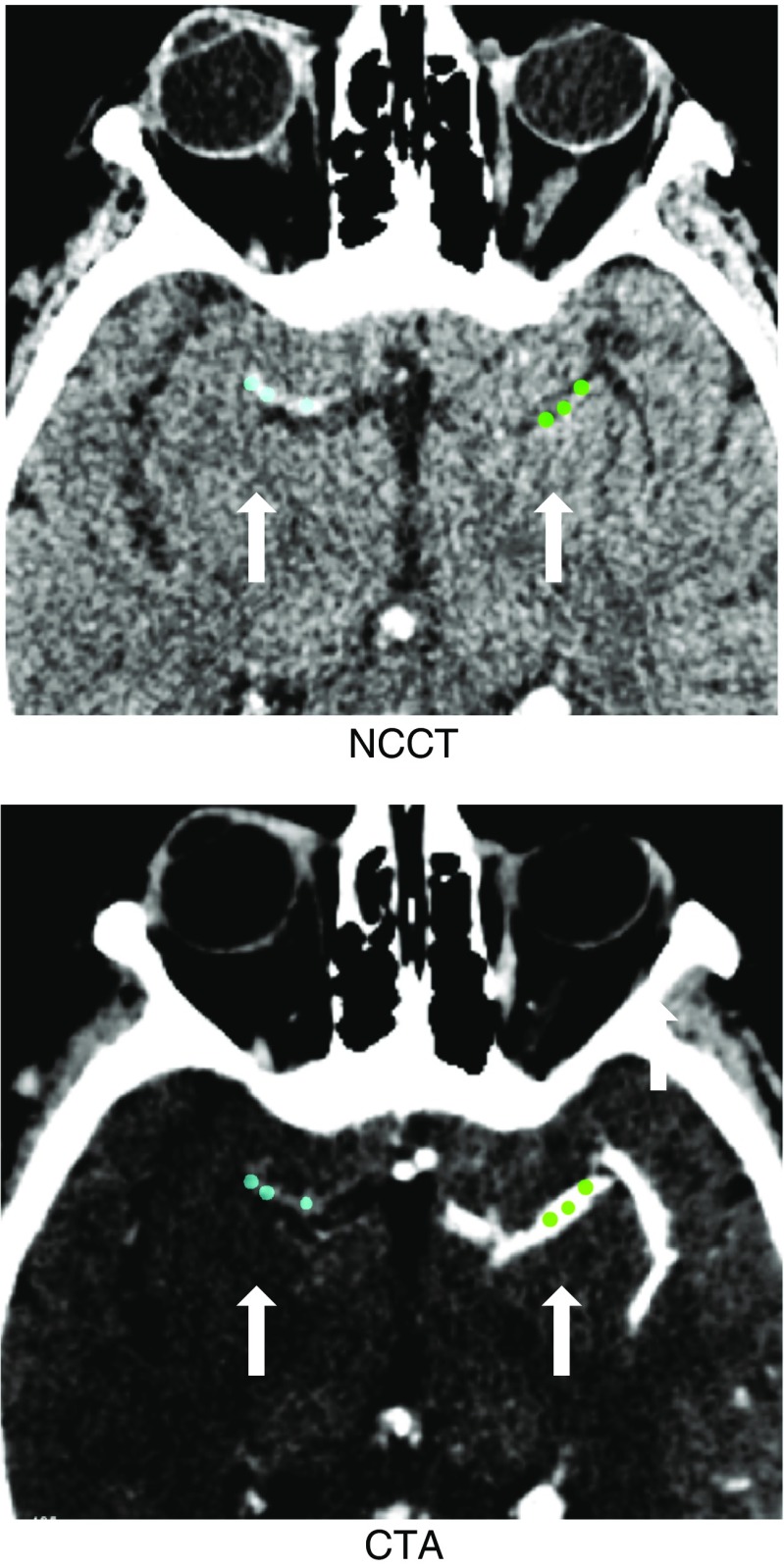
Illustration of the thrombus and contralateral ROI placements on registered NCCT (*top*) and CTA (*bottom*). The *white arrows* are indicating the locations of the thrombus ROIs (*blue spheres*) and the contralateral ROIs (*green spheres*)

Thrombus measurements can be performed in any radiological workstation [3]. This task consists of detecting the thrombus in NCCT images and measuring its density by the placement standardized measurement tools (e.g., ROIs such as 2D ellipses or 3D spheres); see Fig. 1. As a support for the detection of low-density thrombi, CTA images can be displayed simultaneously with the NCCT images. For the simultaneous displaying of NCCT and CTA images, CTA images were automatically aligned with NCCT images using a rigid registration of the open source software Elastix® [13]. In this study, we performed the annotations and measurements with in Mevislab® developed software [14]. Thrombus density was measured in the proximal, middle, and distal part using three separate spherical volumes with a radius of 1 mm. In case of small thrombi, the ROIs often overlapped, which was accepted in our study. Our method assured that in case of overlapping ROIs, the attenuation values of every pixel were counted only once. In a similar fashion, contralateral density measures were performed at the corresponding site of occlusion [3]. The middle ROI density measurement of both the thrombus and contralateral artery was used for the single ROI measurement. Based on the CT attenuation values, the average absolute thrombus density in Hounsfield units (aHU) and average relative thrombus density in Hounsfield units (rHU) for three and one ROIs, abbreviated as aHU3, rHU3, aHU1, and rHU1, respectively, were determined.

Three expert neuroradiologists each with more than 10 years of experience (AY, LB, CM) and two non-expert trained observers (MB and CW, one with a MSc in Biomedical Engineering and a fifth-year student in MSc Technical Medicine) placed the ROIs. The trained observers received approximately 3 h of training. The observers only had access to baseline NCCT and CTA images during the measurement sessions and were blinded from measured intensity, all clinical information, and each other’s measurements.

Before the measurements, a calibration session was organized in which the observers performed the measurement in a training set of nine randomly selected datasets. This calibration session was used to assess any differences in interpretation and measurement strategy. Differences were discussed in a subsequent consensus meeting, after which the following additional instruction was provided; in case of thrombus at a bifurcation, only the longest branch should be used for the measurement.

During the measurements, the observers could exclude a dataset if the image quality was considered insufficient to confidently place the ROIs, or if the occlusion was not distinguishable. If a dataset was excluded, the reason was recorded.

After the measurements, a verification of the number of markers as well as a visual check of all ROIs was performed by a single external observer (ES) to detect missing, supernumerary, or incorrectly labeled ROI. These suspicious ROIs were discussed and corrected by the same observer if necessary. The number of incorrect ROI placements and performed corrections was recorded.

### Statistical analysis

The interobserver agreement of the thrombus density measurements was evaluated by performing a paired comparison of the measurements of observers 1 and 2 and of observers 1 and 3. The accuracy of the trained non-expert observers was assessed by comparing measurements of observers 4 and 5 with that of observer 1 as the reference standard. The interobserver agreement of the trained non-expert observers was assessed by comparing measurements of observer 4 and observer 5. For all comparisons, Bland–Altman analysis was performed and the intraclass correlation coefficient (ICC) was calculated.

To estimate the bias introduced by using only one instead of three ROIs, we evaluated the intermethod agreement by comparing the single ROI measurements with the three ROI measurements, using the latter as reference. Statistical significance of the differences between one and three ROI measurements was tested using paired *t* tests for normally distributed data or a related-sample Wilcoxon signed-rank test otherwise. Furthermore, a Bland–Altman analysis was performed and the ICC was determined.

An ICC superior to 0.80 was considered as excellent, between 0.60 and 0.79 as good, between 0.40 and 0.59 as fair, and below 0.39 as poor. Normality of the distributions was tested using the Shapiro–Wilk test. Significance level was set to a *p* value of 0.05. To identify outliers prior to the ICC calculations, we used Tukey’s hinges method [15], in which observations below Q1 − 1.5 (Q3 − Q1) and above Q3 + 1.5(Q3 − Q1) were excluded. All analyses were performed using IBM SPSS Statistics software, version 20.0 (IBM Corporation, Armonk, NY, USA).

## Results

The average age was 66 (±13) years and 62.1 % (82) of the patients were male. For some patients, the measurements could not be performed. Three observers excluded the dataset of one patient and two observers excluded two datasets of two patients because of insufficient contrast for accurate assessment; one (expert) observer did not identify an occlusion for one patient, and one (trained) observer could not detect an occlusion in three patients. A total of 55 out of 522 annotations (11 %) required a correction during follow-up sessions. Forty-four (8.5 %) out of 522 measures were outliers, consisting of 14 absolute and 30 relative densities. These were excluded in the calculation of the ICCs.

The interobserver agreement for absolute density was good to excellent for the two expert observer pairs with ICCs of 0.81 and 0.91 using three ROIs and 0.47 and 0.74 using one ROI. For relative density, the agreement was poor to good with ICCs of 0.25 and 0.74 for using three ROIs and 0.31 and 0.66 for using one ROI. The width of the limits of agreement for absolute density was smaller using three ROIs than for one ROI (Online Resource 2); the same pattern can be observed for relative density measurements (Online Resource 3).

The agreement of the trained observers with the reference expert observer was good for absolute density measurements with ICCs of 0.71 and 0.79 when using three markers. For a single measurement, the agreement was somewhat lower with ICCs of 0.37 and 0.71. The agreement was poor and fair for relative densities with ICCs ranging between 0.15 and 0.50. Similar to the expert observers, the interobserver agreement is better for the absolute measurement using three markers, with an ICC of 0.71, which was comparable to the expert interobserver agreement. The results of all Bland and Altman analysis and ICC calculations are shown in Table 1. The Bland–Altman graphs are shown in the Online Resource 2 and Online Resource 3 for absolute and relative measures, respectively.

**Table 1:** Bland–Altman analysis and the ICC calculations of the interobserver agreement between expert observers (observers 1 and 2 and observers 1 and 3), interobserver agreement of trained observers (observers 4 and 5), and comparison of the trained observers (4 and 5) with the reference measurements of observer 1

			ICC	*n* (ICCs)	Mean differences	Upper–lower LoA
Expert observers	Observer 1 and 2 (*n* = 71)	aHU3	0.90*	70	0.1	7.1–6.8
		aHU1	0.74*	68	−0.7	10.3–11.7
		rHU3	0.72*	66	−0.10	0.48 - 0.64
		rHU1	0.66*	65	−0.09*	0.46–0.63
	Observer 1 and 3 (*n* = 61)	aHU3	0.54*	61	5.8*	16.1–4.5
		aHU1	0.47*	60	4.9*	21.0–11.2
		rHU3	0.25*	60	0.07	0.64–0.49
		rHU1	0.31*	58	0.04	0.75–0.66
Trained observers	Observer 4 and 5 (*n* = 127)	aHU3	0.70*	123	−1.6	12.3–16.0
		aHU1	0.40*	126	1.2	16.8–14.4
		rHU3	0.48*	123	−0.01	0.56–0.60
		rHU1	0.24*	117	0.01	1.00–0.97
Reference observer with trained observers	Observer 1 and 4 (*n* = 129)	aHU3	0.79*	127	1.4*	10.4–7.6
		aHU1	0.71*	127	1.1	13.3–11.2
		rHU3	0.50*	126	0.03	0.49–0.43
		rHU1	0.46*	120	0.00	0.67–0.68
	Observer 1 and 5 (*n* = 127)	aHU3	0.71*	123	3.0*	15.5–9.5
		aHU1	0.37*	124	2.2*	16.4–12.0
		rHU3	0.46*	123	0.03	0.54–0.50
		rHU1	0.15	116	0.00	0.95–0.93
Accuracy	Expert observers (*n* = 263)	aHU	0.84*	259	−1.0*	7.8–9.8
		rHU	0.69*	252	−0.03*	0.10–0.73

*ICC* intraclass correlation coefficient, *LoA* limit of agreement, *aHU* absolute thrombus density in Hounsfield unit, *rHU* relative thrombus density

*p value *<*.05

The intermethod agreement of the measurements with one ROI compared to the three ROIs was excellent with an ICC of 0.84 for absolute and good with an ICC of 0.69 for relative density measurements. The Bland and Altman graphs are shown in Online Resource 4.

## Discussion

Our study shows that interobserver agreement in thrombus density measurements varies considerably from excellent to poor depending on the method used. Agreement was higher for absolute than for relative density measurements and higher for using three ROIs compared to using one ROI.

The interobserver agreement of the absolute thrombus density measurements by the expert observers is in line with previously reported values [4]. Another study reported a similar reliability for an intraobserver evaluation [3]. Despite the fact that relative density measurements are being used in studies [3, 4], no data on interobserver agreement were previously reported. In most studies, three markers are used [3, 6], a method supported by our analysis, whereas in one study, the number of ROIs was not reported [4]. As shown in our study, density measurement can be prone to errors as a moderate amount of measurements required addition removal or adjustment of a ROI after the inaccuracies were pointed out by an external observer. None of these adjustments have previously been reported in the literature.

The larger variation for relative density measurements suggests that placement of ROIs in the contralateral vessel is less robust than ROI placement in the thrombus. The relative density measurements have been introduced to correct for the hematocrit. However, our findings suggest that this advantage may potentially be negated by the increased variability of thrombus measurement [3, 4].

We have shown that trained non-expert observers have an interobserver agreement similar to expert observers, and a good agreement with expert observer measurements. However, the detection of small occlusions was sometimes more challenging for them and a few of these were missed. Our study indicates a good correlation between three ROIs and one ROI density measurements. However, overall, interobserver agreement was lower when using one ROI compared to three ROIs. Thrombi are generally heterogeneous in composition [16]; therefore, a single ROI assessment samples only a limited region within the thrombus compared to multiple ROIs, rendering the measured aHU and rHU density more sensitive to the specific location of the ROI in the thrombus (i.e., sampling bias). High user variability in measurements may result in a limited reproducibility and contribute to differences in found associations, such as that found in studies assessing the association of thrombus density with patient outcome [5, 6, 10, 11].

This study has a number of limitations. There was a large difference in the interobserver agreement between different observer pairs. Despite the training session, two expert observers routinely selected locations in the thrombus with higher signal intensities than the third expert observer, which resulted in a systematic bias. Thin-slice (≤2.5 mm) NCCT and CTA data were available only for 170 out of 388 patients. Because the trial from which the data were collected was still ongoing, the full dataset was not available at the time of this study. Because the data were coming from a large number of medical centers, imaging parameters such as reconstruction parameters and slice thickness varied considerably. However, since our observers annotated the same images, it is not expected that these variations have a large influence on the interobserver agreement. This study is limited by the absence of a reference standard for the actual histological thrombus characteristics. As such, the accuracy of the density measurements could not be determined. Furthermore, we also could not address the difference in timing because all observers performed the full measurement by placement of six markers. The interobserver variation of NCCT measurements was performed with the support of simultaneous viewing of CTA images. This is not routine in acute care or radiological clinical practice. However, for thrombus density measurement in clinical studies, which is by far the most common application of thrombus density measurements, the support of CTA is performed in most studies; CTA has been used as guidance for the presence and location assessment of the clot location [5, 9, 10], has been used as simultaneous visual support while performing the NCCT density measurement [3, 4, 11], and has been used for the assessment of the clot burden after an affine registration of CTA and NCCT images [12]. The visual support of CTA may result in an increase in interobserver agreement, since placement of annotations in low-density thrombi in NCCT is considered difficult. Finally, we have used the middle of the three ROIs was used as the single ROI. This may have resulted in an overestimation of the actual agreement compared to an evaluation in which the single ROI measurement would have been performed separately.

With an increasing number of available treatment options, precise characterization of the target thrombus is required to enable optimal treatment selection [17]. Multiple factors for treatment efficacy prediction are currently investigated, including thrombus location [18], thrombus length [19], enhancement in thrombus [20], collaterals status [21], and thrombus density. Nevertheless, all possess only limited associations, for example, recent research showed that thrombus length measured on NCCT (>8 mm) is a potential predictor of intra-venous treatment failure [3]. However, commonly smaller thrombi do not dissolve after intra-venous treatment administration. Investigating and improving understanding of the thrombus composition will provide additional information to support optimized treatment decision. In this process, estimations of measurement accuracy and reproducibility are mandatory. This study has contributed in thrombus measurement accuracy estimations by assessing the interobserver agreement for different measurement methods.

## Conclusions

Our study reveals that the interobserver agreement for absolute density measurement is superior to relative density measurement. The use of multiple regions of interest when assessing density provides a more reliable measurement compared to single sample measurements. Absolute thrombus density can reliably be measured even by non-expert observers using multiple ROIs; however, a few patients with small indistinct thrombi would require support from experts.

### Electronic supplementary material

ESM 1 (PDF 1277 kb)

## Appendix

### List of MR CLEAN investigators and affiliations

Olvert A. Berkhemer, M.D.,^1,2^ Puck S.S. Fransen, M.D.,^2,3^ Debbie Beumer, M.D.^2,4^ Lucie A. van den Berg, M.D.,^5^ Hester F. Lingsma, M.D., Ph.D.,^7^ Albert J. Yoo, M.D.,^8^ Wouter J. Schonewille, M.D.,^9^ Jan Albert Vos, M.D., Ph.D.,^10^ Paul J. Nederkoorn, M.D., Ph.D.,^5^ Marieke J.H. Wermer, M.D., Ph.D.,^11^ Marianne A.A. van Walderveen, M.D., Ph.D.,^12^ Julie Staals, M.D., Ph.D.,^4^ Jeannette Hofmeijer, M.D., Ph.D.,^13^ Jacques A. van Oostayen, M.D., Ph.D.,^14^ Geert J. Lycklama à Nijeholt, M.D., Ph.D.,^15^ Jelis Boiten, M.D., Ph.D.,^16^ Patrick A. Brouwer, M.D.,^3^ Bart J. Emmer, M.D., Ph.D.,^3^ Sebastiaan F. de Bruijn, M.D., Ph.D.,^17^ Lukas C. van Dijk, M.D.,^18^ L. Jaap Kappelle, M.D., Ph.D.,^19^ Rob H. Lo, M.D.,^20^ Ewoud J. van Dijk, M.D., Ph.D.,^21^ Joost de Vries, M.D., Ph.D.,^22^ Paul L.M. de Kort, M.D., Ph.D.,^23^ Jan S.P. van den Berg, M.D., Ph.D.,^24^ Boudewijn A.A.M. van Hasselt, M.D.,^25^ Leo A.M. Aerden, M.D., Ph.D.,^26^ René J. Dallinga, M.D.,^27^ Marieke C. Visser, M.D., Ph.D.,^28^ Joseph C.J. Bot, M.D., Ph.D.,^29^ Patrick C. Vroomen, M.D., Ph.D.,^30^ Omid Eshghi, M.D., ^31^ Tobien H.C.M.L. Schreuder, M.D.,^32^ Roel J.J. Heijboer, M.D.,^33^ Koos Keizer, M.D., Ph.D.,^34^ Alexander V. Tielbeek, M.D., Ph.D.,^35^ Heleen M. den Hertog, M.D., Ph.D.,^36^ Dick G. Gerrits, M.D., ^37^ Renske M. van den Berg-Vos, M.D., Ph.D.,^38^ Giorgos B. Karas, M.D.,^39^ Ewout W. Steyerberg, M.D., Ph.D.,^7^ H. Zwenneke Flach, M.D.,^26^ Henk A. Marquering Ph.D.,^40,1^ Marieke E.S. Sprengers, M.D., Ph.D.,^1^ Sjoerd F.M. Jenniskens, M.D., Ph.D.,^41^ Ludo F.M. Beenen, M.D.,^1^ René van den Berg, M.D., Ph.D.,^1^ Peter J. Koudstaal, M.D., Ph.D.,^2^ Wim H. van Zwam, M.D., Ph.D.,^6^ Yvo B.W.E.M. Roos, M.D., Ph.D., ^5^ Aad van der Lugt, M.D., Ph.D., ^3^ Robert J. van Oostenbrugge, M.D., Ph.D., ^4^ Charles B.L.M. Majoie, M.D., Ph.D., ^1^ and Diederik W.J. Dippel, M.D., Ph.D. ^2^

1 Department of Radiology, Academic Medical Center Amsterdam, the Netherlands (AMC); 2 Department of Neurology, Erasmus MC University Medical Center Rotterdam, the Netherlands; 3 Department of Radiology, Erasmus MC University Medical Center Rotterdam, the Netherlands; 4 Department of Neurology, Maastricht University Medical Center and Cardiovascular Research Institute Maastricht (CARIM), the Netherlands; 5 Department of Neurology, Academic Medical Center Amsterdam, the Netherlands; 6 Department of Radiology, Maastricht University Medical Center, the Netherlands; 7 Department of Public Health, Erasmus MC University Medical Center Rotterdam, the Netherlands; 8 Department of Radiology, Texas Stroke Institute, Texas, United States of America; 9 Department of Neurology, Sint Antonius Hospital, Nieuwegein, the Netherlands; 10 Department of Radiology, Sint Antonius Hospital, Nieuwegein, the Netherlands; 11 Department of Neurology, Leiden University Medical Center, the Netherlands; 12 Department of Radiology, Leiden University Medical Center, the Netherlands; 13 Department of Neurology, Rijnstate Hospital, Arnhem, the Netherlands; 14 Department of Radiology, Rijnstate Hospital, Arnhem, the Netherlands; 15 Department of Radiology, MC Haaglanden, the Hague, the Netherlands; 16 Department of Neurology, MC Haaglanden, the Hague, the Netherlands; 17 Department of Neurology, HAGA Hospital, the Hague, the Netherlands; 18 Department of Radiology, HAGA Hospital, the Hague, the Netherlands; 19 Department of Neurology, University Medical Center Utrecht, the Netherlands; 20 Department of Radiology, University Medical Center Utrecht, the Netherlands; 21 Department of Neurology, Radboud University Medical Center, Nijmegen, the Netherlands; 22 Department of Neurosurgery, Radboud University Medical Center, Nijmegen, the Netherlands; 23 Department of Neurology, Sint Elisabeth Hospital, Tilburg, the Netherlands; 24 Department of Neurology, Isala Klinieken, Zwolle, the Netherlands; 25 Department of Radiology, Isala Klinieken, Zwolle, the Netherlands; 26 Department of Neurology, Reinier de Graaf Gasthuis, Delft, the Netherlands; 27 Department of Radiology, Reinier de Graaf Gasthuis, Delft, the Netherlands; 28 Department of Neurology, VU Medical Center, Amsterdam, the Netherlands; 29 Department of Radiology, VU Medical Center, Amsterdam, the Netherlands; 30 Department of Neurology, University Medical Center Groningen, the Netherlands; 31 Department of Radiology, University Medical Center Groningen, the Netherlands; 32 Department of Neurology, Atrium Medical Center, Heerlen, the Netherlands; 33 Department of Radiology, Atrium Medical Center, Heerlen, the Netherlands; 34 Department of Neurology, Catharina Hospital, Eindhoven, the Netherlands; 35 Department of Radiology, Catharina Hospital, Eindhoven, the Netherlands 36 Department of Neurology, Medical Spectrum Twente, Enschede, the Netherlands; 37 Department of Radiology, Medical Spectrum Twente, Enschede, the Netherlands; 38 Department of Neurology, Sint Lucas Andreas Hospital, Amsterdam, the Netherlands; 39 Department of Radiology, Sint Lucas Andreas Hospital, Amsterdam, the Netherlands; 40 Department of Biomedical Engineering and Physics, Academic Medical Center Amsterdam, the Netherlands; 41 Department of Radiology, Radboud University Medical Center, Nijmegen, the Netherlands

### MR CLEAN Acknowledgements & funding

The MR CLEAN trial was funded by the Dutch Heart Foundation and through unrestricted grants from AngioCare BV, Covidien/EV3®, MEDAC Gmbh/LAMEPRO, and Penumbra Inc.

We thank the following persons for their help and advice during the conduct of MR CLEAN:

### Data monitoring and safety board

Chair: Martin M. Brown, National Hospital for Neurology & Neurosurgery, London, UK. Member: Thomas Liebig, Med. Fakultät, Univ Köln, Germany, Independent Statistician: Theo Stijnen, Leiden University Medical Center, Leiden, the Netherlands

### Advisory board

Tommy Andersson, neuro interventionist, Karolinska Univeristy Hospital, Stockholm, Sweden, Heinrich Mattle, neurologist, University hospital, Bern, Switzerland, Nils Wahlgren, neurologist, Karolinska Hospital, Stockholm, Sweden.

### Research nurses/local trial coordinators

Esther van der Heijden, Naziha Ghannouti; Erasmus MC University Medical Center Rotterdam, the Netherlands. Nadine Fleitour, Imke Hooijenga; Academic Medical Center Amsterdam, the Netherlands. Corina Puppels, Wilma Pellikaan; Sint Antonius Hospital, Nieuwegein, the Netherlands. Annet Geerling; Radboud University Nijmegen Medical Center, the Netherlands. Annemieke Lindl-Velema; Maastricht University Medical Center, the Netherlands. Gina van Vemde; Isala Klinieken, Zwolle, The Netherlands. Ans de Ridder, Paut Greebe; University Medical Center Utrecht, the Netherlands. José de Bont-Stikkelbroeck; Sint Elisabeth Hospital, Tilburg, the Netherlands. Joke de Meris; MC Haaglanden, the Hague, the Netherlands. Kirsten Janssen; Leiden University Medical Center, the Netherlands. Willy Struijk; HAGA Hospital, the Hague, the Netherlands. Tiny Simons; Atrium MC, Heerlen, the Netherlands. Gert Messchendorp, Friedus van der Minne; University Medical Center Groningen, the Netherlands. Hester Bongenaar; Catharina Hospital, Eindhoven, the Netherlands.

### PhD/Medical students

Silvan Licher, Nikki Boodt, Adriaan Ros, Esmee Venema, Ilse Slokkers, Raymie-Jayce Ganpat, Maxim Mulder, Nawid Saiedie, Alis Heshmatollah, Stefanie Schipperen, Stefan Vinken, Tiemen van Boxtel, Jeroen Koets; Erasmus MC University Medical Center Rotterdam, the Netherlands. Merel Boers, Emilie Santos, Jordi Borst, Ivo Jansen, Manon Kappelhof, Marit Lucas, Ralph Geuskens, Renan Sales Barros, Roeland Dobbe, Marloes Csizmadia; Academic Medical Center Amsterdam, the Netherlands.

## Abbreviations

ICC: Intraclass correlation coefficients
aHU: Absolute thrombus density in Hounsfield unit
rHU: Relative thrombus density
HU: Hounsfield unit
LoA: Limits of Agreement
SD: Standard deviation
