# Supplementary material for: Observer variability of absolute and relative thrombus density measurements in patients with acute ischemic stroke
Source: Neuroradiology. 2015 Oct 22;58:133–9. doi: 10.1007/s00234-015-1607-4 (PMC4773501; doi:10.1007/s00234-015-1607-4)

Electronic Supplementary material for

Observer variability of absolute and relative thrombus density measurements in patients with acute ischemic stroke

Online Resource 1. Table of the information details on scanners manufacturers, models, CT parameters such as tube voltage, convolution kernel, as well as reconstructions modalities such as slice thickness and filter.

		n patients
**Scanner manufacturer and model**	**GE MEDICAL SYSTEMS**	**13**
	LightSpeed VCT	13
	**PHILIPS**	**24**
	Brilliance 40	10
	Brilliance 64	5
	iCT 256	9
	**SIEMENS**	**53**
	Biograph 64	2
	Sensation 64	22
	SOMATOM Definition AS+	4
	SOMATOM Definition Flash	25
	**TOSHIBA**	**42**
	Aquilion ONE	42
**Tube voltage in KVp**	100	2
	120	130
**Slice thickness reconstruction in mm**	≤0.5	74
	≤0.75<	25
	≤1<	26
	≤1.5<	7
**Convolution kernels types**	FCXX*	19
	FLXX*	23
	HXXx*	45
	J45s\X*	8
	SOFT	13
	UA	2
	UB	22
**Filter types**	none	29
	FLAT	29
	LARGE	23
	MEDIUM	19
	SMALL	8
	UA	2
	UB	22

*Kernel type details: FC22 , FC25 , FC26 , FC27 , FC28 , FC29 , FC30 , FC31 , FC32 , FC33 , FC34 , FC35 , FC36 , FC69 , FC70 , FC71 , FC72 , FL01 , FL02 , FL03 , FL04 , FL05 , FL06 , FL07 , FL08 , FL09 , FL10 , FL11 , FL12 , FL13 , FL14 , FL15 , FL16 , FL17 , FL18 , FL19 , FL20 , FL21 , FL22 , FL23 , H30f , H31s , H41s , H60s , H70h , J45s\10 , J45s\11 , J45s\4 , J45s\5 , J45s\6 , J45s\7 , J45s\8 , J45s\9.

Online Resource 2. Bland-Altman plots of the absolutes density measurements. For all plots, results using thee ROIs are shown in the left column, and results using one ROI are displayed in the right column. The upper row shows the comparison of expert observers (observer 1 and 2 in blue and observer 1 and 3 in green). The middle row shows the comparison of the reference expert observer with trained observer 4 in orange and with trained observer 5 in purple. The lower row shows the reliability of trained observers (observer 4 and 5). The dotted lines represent the limits of agreements; the solid line represents the mean paired difference.

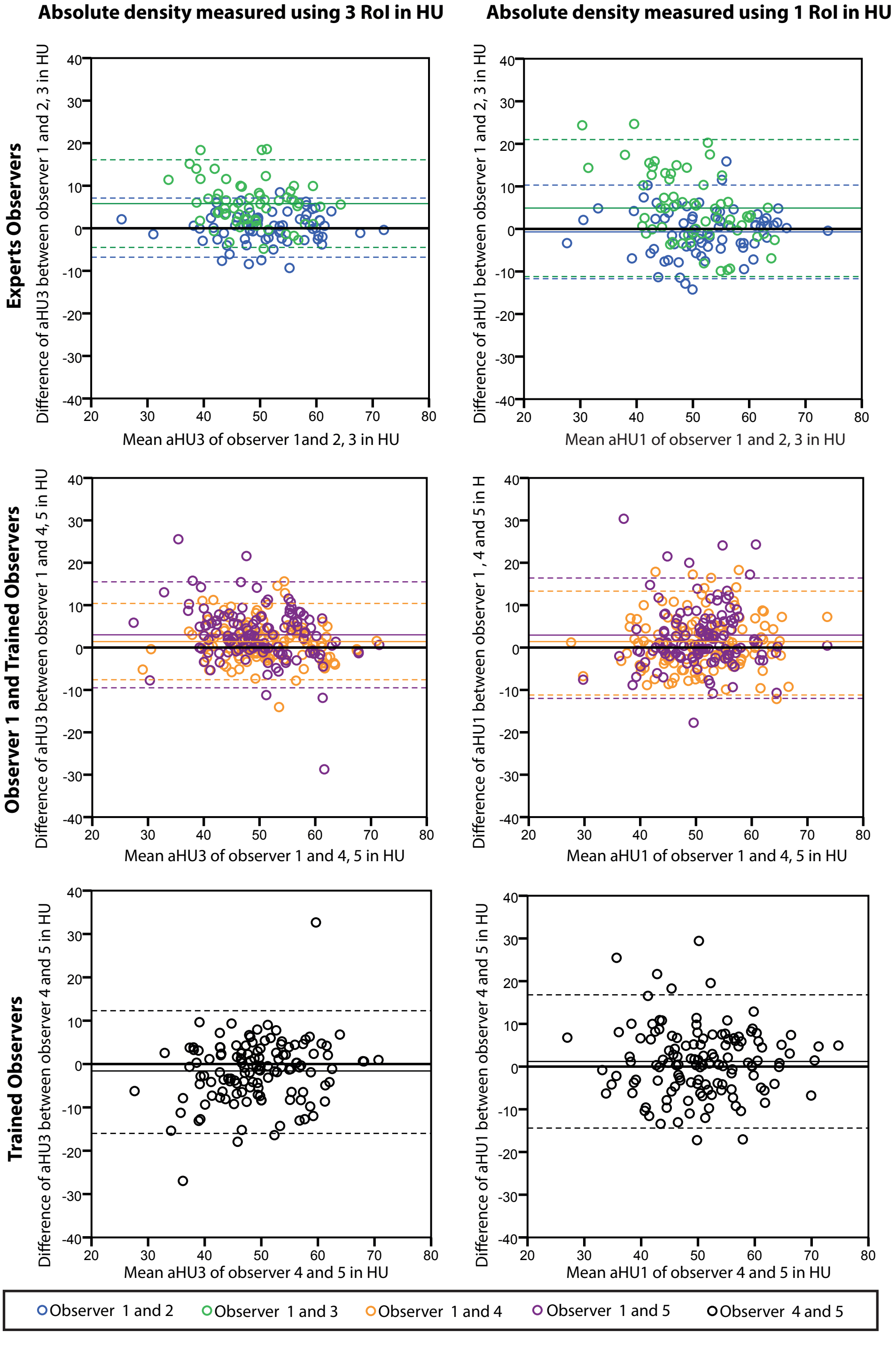

Online Resource 3. Bland-Altman plots of the relatives density measurements. For all plots, results using thee ROIs are shown in the left column, and results using one ROI are displayed in the right column. The upper row shows the comparison of expert observers (observer 1 and 2 in blue and observer 1 and 3 in green). The middle row shows the comparison of the reference expert observer with trained observer 4 in orange and with trained observer 5 in purple. The lower row shows the reliability of trained observers (observer 4 and 5). The dotted lines represent the limits of agreements; the solid line represents the mean paired difference.

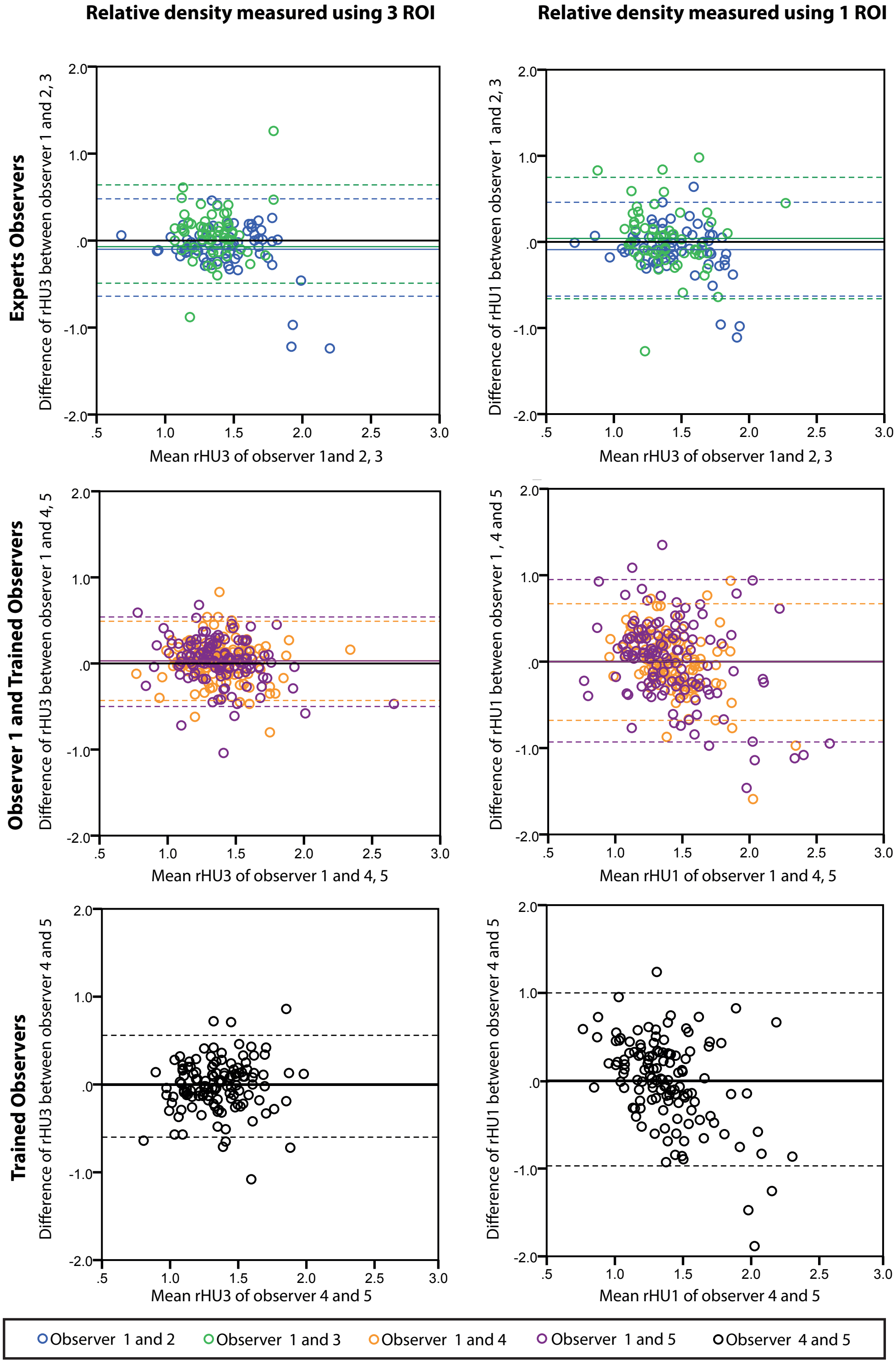

Online Resource 4. Bland-Altman plots of the comparison of the 1 ROI and 3 ROIs thrombus density measurements by expert observers. The upper frame shows the results for absolute density measurements and the lower frame shows the results of the relative density measurements. The dotted lines represent the limits of agreements; the solid line represents the mean difference.

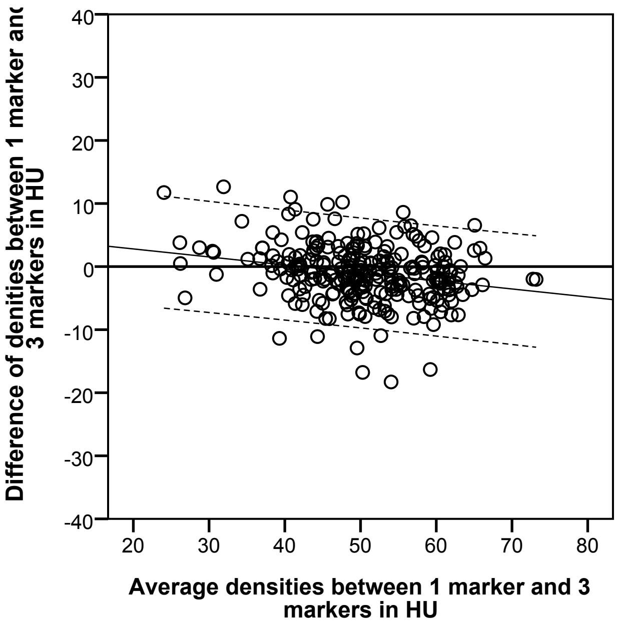

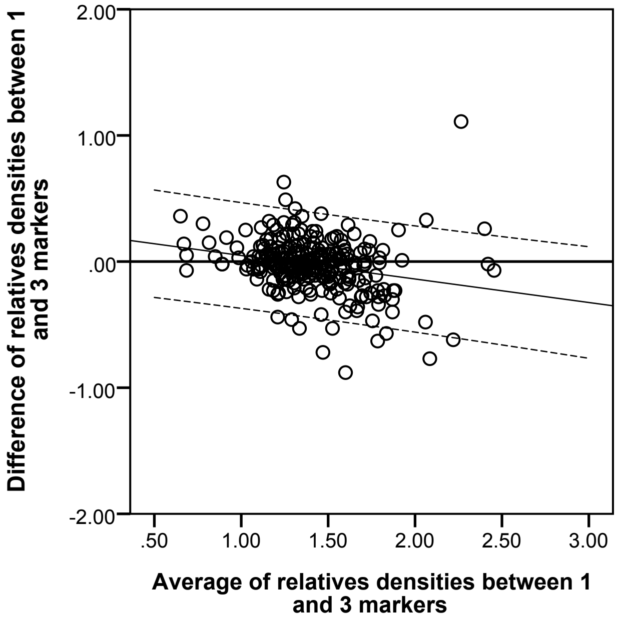

Supplement: Supplementary file 1 — (PDF 1277 kb) [file 234_2015_1607_MOESM1_ESM.pdf]
